# Comparative evaluation of ELISA, PPIA, and HPLC/MS for microcystin quantification in seven lakes of western Michigan

**DOI:** 10.3389/fmicb.2026.1733970

**Published:** 2026-05-21

**Authors:** Chen Cheng, Xiaonan Tang, Yongjiu Cai, Dailan Deng, Liqiang Xie, Richard R. Rediske

**Affiliations:** 1 State Key Laboratory of Lake and Watershed Science for Water Security, Nanjing Institute of Geography and Limnology, Chinese Academy of Sciences, Nanjing, China; 2 Department of Civil and Environmental Engineering, The George Washington University, Washington, DC, United States; 3 School of Ecology and Environment, Anhui Normal University, Wuhu, Anhui, China; 4 Annis Water Resource Institute, Grand Valley State University, Muskegon, MI, United States

**Keywords:** algal blooms, ELISA, HPLC/MS, microcystins, PPIA

## Abstract

Cyanobacterial harmful algal blooms (cHABs) pose escalating risks to aquatic ecosystems and public health through the release of microcystins (MCs), emphasizing the need for accurate detection and quantification. This study compared three widely used MC detection methods—enzyme-linked immunosorbent assay (ELISA), protein phosphatase inhibition assay (PPIA), and high-performance liquid chromatography coupled with mass spectrometry (HPLC/MS)—across about 200 samples from seven drowned river mouth lakes in western Michigan, USA. Results revealed that PPIA and ELISA overestimated the concentration of MC-LR. Specifically, the measurement error of MC-LR equivalent by ELISA and PPIA increased at low concentrations (<0.15 μg/L HPLC/MS-quantified MC-LR) but remained relatively stable at higher levels. Further analysis revealed that among the MC congeners, MC-RR showed the highest contribution rate (60%) to the measurement error by ELISA, while MC-YR showed the highest contribution rate (69%) for PPIA. These discrepancies correlated strongly (*p* < 0.001) with cyanobacterial composition (notably *Microcystis* dominance), bloom intensity, and lake trophic status. Method selection should therefore consider congener composition, community structure, and nutrient conditions. A tiered analytical framework—using ELISA for rapid screening, PPIA for bioactivity assessment, and HPLC/MS for confirmatory quantification—offers a robust approach for reliable toxin monitoring, while integration with watershed nutrient management can further mitigate cHAB risks and protect freshwater ecosystem health.

## 1 Introduction

The global intensification of eutrophication has led to more frequent and severe cyanobacterial harmful algal blooms (cHABs), particularly in freshwater systems where nutrient enrichment and climate change synergistically promote bloom persistence (Tang et al., 2022; Yan et al., 2017). *Microcystis* species, as dominant bloom-formers in many freshwater lakes, disrupt ecological balance and produce microcystins (MCs), a group of potent hepatotoxins of major public health concern (Cheng et al., 2023b). MCs are the most prevalent algal toxins in temperate lakes, largely retained within cyanobacterial cells during active growth (Cheng et al., 2023a; Tang et al., 2024). When blooms decay or undergo cell lysis, these intracellular toxins are released into the water column, inhibiting protein phosphatases 1 and 2A and causing downstream cytotoxicity (Cheng et al., 2023b). Accurate quantification of these toxins is therefore critical for assessing ecological risks, protecting public health, and informing lake management policies.

Several analytical techniques are commonly employed for cyanotoxin quantification, including enzyme-linked immunosorbent assay (ELISA), protein phosphatase inhibition assay (PPIA), and high-performance liquid chromatography coupled with mass spectrometry (HPLC/MS) (Adams et al., 2025; Meriluoto et al., 2017; Preece et al., 2017; U.S. EPA, 2015a,b, 2016a,b). These methods rely on distinct detection principles: immunochemical recognition for ELISA, enzymatic inhibition for PPIA, and physicochemical separation coupled with mass detection for HPLC/MS. Each approach offers unique advantages but also distinct limitations. For MCs, ELISA allows rapid and cost-effective screening based on antibody binding, but cross-reactivity among congeners and the inability to distinguish free and conjugated forms limit its specificity (Preece et al., 2021; McElhiney and Lawton, 2005; Mountfort et al., 2005). PPIA measures total bioactivity by assessing inhibition of PP1 activity, providing a rapid and low-cost estimate of overall toxicity, though variations in inhibition strength across congeners hinder congener-specific quantitation (Kumar et al., 2020; Tillmanns et al., 2007). Although liquid chromatography–tandem mass spectrometry (LC–MS/MS) operating in multiple reaction monitoring (MRM) mode has become the dominant analytical platform for the identification and quantification of microcystins, HPLC–MS was widely adopted in earlier microcystin research and monitoring programs. This technique enables congener-specific identification and quantification with high chemical specificity and analytical sensitivity by separating individual microcystin congeners according to their chromatographic behavior and molecular mass. Consequently, HPLC–MS has long been regarded as a confirmatory analytical technique for cyanotoxin analysis and is commonly used as the benchmark method in environmental monitoring studies. Both methods require sophisticated instrumentation and trained personnel, and its application is further limited by the lack of widely available certified standards (Hu, 2022; McElhiney and Lawton, 2005; Tillmanns et al., 2007; Triantis et al., 2010). Consequently, differences in congener composition, sample matrix effects, and toxin conjugation state can lead to inter-method discrepancies and inconsistent toxin assessments.

Despite extensive use of these techniques, most prior comparison studies have been constrained by small sample sizes or artificial conditions (Kumar et al., 2020). Many investigations compared only two methods, relied on spiked water or laboratory-cultured cyanobacteria, or analyzed standardized toxin mixtures (Triantis et al., 2010; Rapala et al., 2002). These controlled systems facilitate method calibration but fail to capture the complex toxin profiles, matrix interferences, and environmental variability characteristic of natural lake systems. Furthermore, existing field-based comparisons typically covered only one or two sites, providing limited representativeness and generalizability (Guo et al., 2017; Tillmanns et al., 2007). Despite these methodological advances, the real-world accuracy and applicability of these analytical approaches under varying eutrophication conditions remain poorly characterized. To address this gap, large-scale, multi-lake evaluations based on field-collected samples are urgently needed.

Western Michigan offers an ideal setting for such investigation. The region hosts numerous drowned river mouth lakes—transitional systems connecting rivers to Lake Michigan—that are heavily used for recreation, fisheries, and tourism but suffer from recurrent cyanobacterial blooms and nutrient enrichment. In this study, seven representative drowned river mouth lakes—Spring Lake, Bear Lake, Mona Lake, Lake Macatawa, Muskegon Lake, White Lake, and Duck Lake—were investigated. Collectively, these lakes serve tens of thousands of residents and visitors annually and encompass a wide eutrophication gradient from mesotrophic to hypereutrophic conditions, providing a realistic and spatially extensive framework for evaluating analytical method performance in natural environments. To capture bloom variability across spatial and temporal gradients, we utilized archived field samples collected between July and August 2006, when cyanobacterial blooms were re-emerging as a major environmental and public health issue in the Great Lakes region, yet unified federal guidance for cyanotoxin monitoring had not yet been established in the United States (Steffen et al., 2014). Although the dataset was generated in 2006, it was not previously developed into a standalone study focused on method comparison. Given the continued occurrence of cyanobacterial blooms and nutrient-related water-quality impairments in western Michigan [Michigan Department of Environment, Great Lakes, and Energy (EGLE), 2024], revisiting this dataset provides a valuable opportunity to establish a baseline and to compare analytical methods under consistent field conditions. By revisiting this large-scale, field-based dataset, the present study aims to elucidate how different detection principles translate into variable toxin estimates under real-world conditions, identify potential systematic biases embedded in early measurements, and enhance the continuity and comparability of long-term cyanotoxin surveillance. Accordingly, ELISA, PPIA, and HPLC/MS were applied to quantify microcystins across the seven lakes, compare inter-method variability and its implications for risk assessment, and evaluate the contribution of congener composition to MC-LR quantification bias. The results provide field-based and historically informed insights that strengthen methodological interpretation, inform monitoring strategy design, and support standardized cyanotoxin assessment across eutrophication gradients.

## 2 Materials and methods

### 2.1 Sampling area

**Figure 1:**
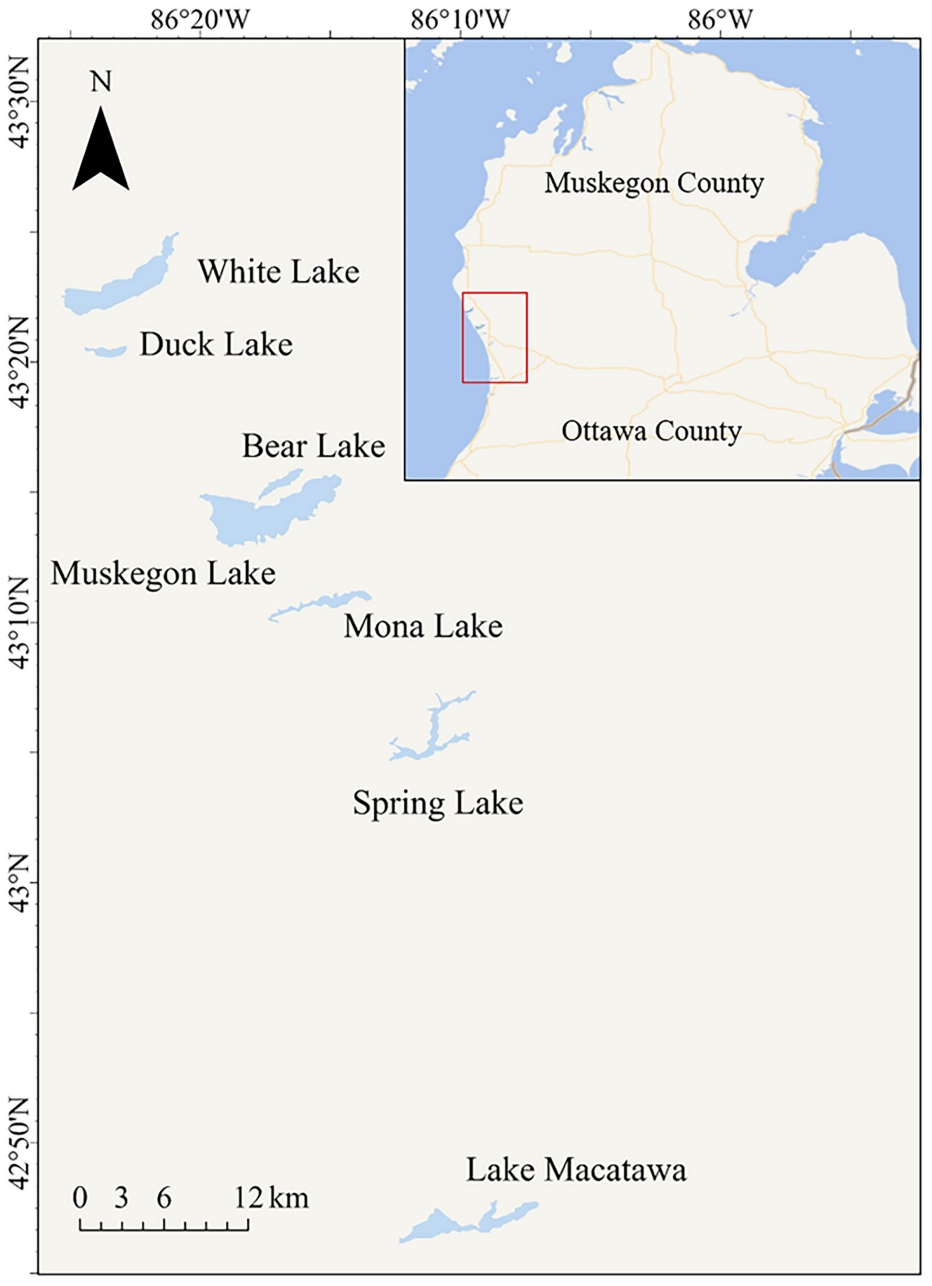
Lakes selected for cyanobacterial blooms monitoring.

Seven drowned river mouth lakes located in western Michigan, USA, namely Spring Lake, Bear Lake, Mona Lake, Lake Macatawa, Muskegon Lake, White Lake, and Duck Lake were investigated from July to August 2006 in this study (Figure 1). Although these lakes are hydrologically influenced by Lake Michigan through direct or indirect connections, they differ substantially in watershed characteristics, nutrient loading, residence time, and bloom dynamics, exhibiting distinct trophic statuses: Spring Lake, Bear Lake, Mona Lake, and Lake Macatawa are classified as hypereutrophic systems, whereas Muskegon Lake, White Lake, and Duck Lake are characterized as mesotrophic to eutrophic systems. The trophic conditions of these lakes have been exacerbated by the invasion of zebra mussels (*Dreissena polymorpha*), which have altered nutrient cycling and increased the frequency and severity of cyanobacterial harmful algal blooms (cHABs) (Nogaro and Steinman, 2014). The proliferation of cyanobacteria in these lakes leads to the production of various cyanotoxins, particularly microcystins (MCs), posing significant ecological and human-health risks.

To assess these impacts, 1 L water samples were collected from each lake using a Schindler sampler. Each of the seven lakes was investigated four times, twice in July and twice in August. Existing studies have demonstrated that the occurrence of algal blooms in this research area exhibits temporal dependence (Hong et al., 2006; Xie et al., 2016). During each investigation, seven specific sampling sites were selected for each lake—four from open-water areas and three are located near the beach—yielding a total of 196 samples from both investigations. After collection, samples were immediately stored at 4 °C in the dark during transport and were processed shortly after arrival at the laboratory. All toxin analyses (ELISA, PPIA, and HPLC/MS) were completed within two weeks of sample collection, therefore, significant toxin degradation during the short period of sample handling and preparation is unlikely.

### 2.2 Sample preparation for toxin analysis

Intracellular MC content was used as an indicator of the potential ecological risks of cyanobacterial blooms. The extraction followed a modified method of Fastner et al. (1998). Briefly, each sample (50–200 mL) was filtered in triplicate to ensure extraction reproducibility through 25 mm Whatman GF/F glass microfiber filters (Cat # 09–874-64, Fisher Scientific, USA). Filters were folded, placed into 2.0 mL microcentrifuge tubes, and stored at 4 °C. *Prior* to extraction, samples were lyophilized using a FREEZONE6 lyophilizer (Labconco, USA). The lyophilized filters were then transferred to separate 15 mL glass vials, and each was treated with 3.0 mL of 75% aqueous methanol, followed by water-bath sonication (Bransonic 5,200, Branson, USA) for 45 min. Samples were centrifuged at 4 °C for 15 min using a Marathon 3,000 centrifuge (Fisher Scientific, USA), and the supernatant (extract) was transferred to a graduated glass centrifuge tube and stored at 4 °C. The remaining filter was treated with an additional 3.0 mL of 75% aqueous methanol, frozen overnight, and then sonicated again for 45 min the following day. After centrifugation, the two extracts were combined, evaporated to dryness under a gentle nitrogen stream on a hot-water bath, and reconstituted in 1 mL methanol followed by 1 mL deionized water. The reconstituted extract (2 mL total) was divided equally: 1 mL for HPLC/MS analysis and 1 mL for ELISA and PPIA testing. All extracts were stored in 2 mL HPLC vials at 4 °C in the dark until analysis.

### 2.3 Determination of microcystin LR equivalents by ELISA

Microcystins were quantified using commercially available 96-well QuantiPlate® ELISA test kits (EnviroLogix Inc., Portland, ME) following the manufacturer’s instructions. This kit employs a competitive ELISA with a measurement range of 0.16 to 2.5 μg/L and uses MC-LR as the test standard. To ensure that sample concentrations fell within the assay’s detection range and to reduce methanol interference, all samples were diluted 10-fold and 100-fold with pyrogen-free deionized water. The residual methanol does not interfere with the assays (see section S1 in Supplementary material). Each sample, along with negative control and three calibrator standards (2.5 μg/L, 0.6 μg/L, and 0.16 μg/L) was analyzed in triplicate. A semi-log calibration curve constructed from these standards was used to determine microcystin concentrations in the unknown samples. Reagents and samples were added sequentially to the 96-well plate and processed according to the instructions. The optical density (OD) of the 96-well plate was measured at a wavelength of 450 nm using a microplate reader (BioTek, Winooski, USA). Quality-control procedures, including triplicate measurements, calibration standards, and routine blank/standard verification, were applied throughout all ELISA analyses.

### 2.4 Determination of microcystin LR equivalents by PPIA

PP1 activity was determined by measuring the release of *p-*nitrophenol from *p-*nitrophenol phosphate, using a modified PPIA protocol (Carmichael and An, 1999) adapted from Satchwell and Boyer (personal communication). This assay was conducted in a 96-well microtitre plate (Cat # 3070, Falcon, USA) with readings taken at a wavelength of 404 nm using a plate reader. The test was performed by adding 10 μL of 50% methanol (MeOH) to blank and control wells, 10 μL of MC-LR standard solution, or 10 μL of each sample (dissolved in 50% aqueous MeOH). Subsequently, 90 μL of Solution D was added, while 90 μL of PP1 solution (New England Biolabs, cat. no. P0754L, diluted 1:800) was added to standard, control, and unknown wells. After a 5-min pre-incubation at 37 °C, 100 μL of PNPP (cat. no. ICN980701, Fisher Scientific, USA) substrate solution was added to each well. Initial readings (t = 0) were taken immediately, followed by a 60-min incubation at 37 °C. Final readings (t = 60) were taken after incubation to measure color production. All environmental samples, a negative control, a positive control, and six calibrators (40 μg/L, 20 μg/L, 12 μg/L, 6 μg/L, 0.6 μg/L and 0.2 μg/L) were analyzed in duplicate. A standard curve was created using the percentage of control activity plotted against the concentrations of the calibrators. The percentage of control activity was calculated as V(standard) - V(blank) / V(control) - V(blank), where V (reaction rate) was determined by final abs/assay time - initial abs/assay time. The part of the curve between 20 and 80% activity corresponding to 0.2 and 12 μg/L MC-LR, respectively. The RSD of inhibition rates was less than 10% (n = 7). Thus 0.2 μg/L was considered as limit of quantification in this study. Some samples were diluted 10-fold and 100-fold with deionized water to ensure concentrations were within the range of detection for the PPIA test. A methanol-interference test was also performed for the PPIA method, and no significant effect was observed at 5% (v/v) methanol (see Section S1 in Supplementary material).

### 2.5 Determination of microcystin LR, YR, RR, and LA by HPLC/MS

In North American lakes, including Bear Lake and Muskegon Lake, MC-LR, MC-YR, MC-RR and MC-LA have been identified as the predominant congeners (Xie et al., 2011; Xie et al., 2016). Across diverse freshwater systems worldwide, these four congeners remain among the most abundant microcystins identified (Chaffin et al., 2023; Yu et al., 2025). Therefore, these congeners were selected as the main detected MC congeners. Microcystins analysis was performed using a Thermo Surveyor MSQ Single Quadrupole Mass Selective Detector and Thermo Spectrasystem HPLC system according to a modified method described by Barco et al. (2002). The measurement range of this method was 1–200 μg/L, and seven concentration levels (1, 5, 10, 20, 50, 100, and 200 μg/L) of the four target MC congeners were used as calibration standards. Microcystin compounds MC-LR, MC-RR, and MC-YR and MC-LA were separated and detected using a Phenomenex Gemini 150 mm column (Los Angeles, USA). A tertiary gradient program was employed to separate both arginine and non-arginine-containing microcystins. The mobile phase consisted of three components: (A) HPLC water, (B) acetonitrile, and (C) 0.10% formic acid in acetonitrile. The gradient program was as follows: 0–1 min (90% A, 10% C), 8–13 min (5% A, 85% B, 10% C), and 14–21 min (90% A, 10% C). The flow rate was maintained at 0.4 mL/min. The MS was operated in the Single Ion Monitoring Mode (SIM), with separate sets of optimized transitions and instrument parameters for arginine-containing and non-arginine-containing microcystin congeners. Quality-control procedures for HPLC/MS included the use of an external standards methods to enhance the accuracy and reliability of quantification. Method performance was evaluated across several representative matrices, with average recoveries of 110.98% for MC-LR, 94.08% for MC-YR, 118.43% for MC-RR, and 89.01% for MC-LA. For each congener, six consecutive injections showed relative standard deviations (RSDs) below 5%, indicating good instrument precision. Blanks and calibration standards were analyzed after every tenth sample to evaluate potential carryover, matrix-induced signal variation, and instrument drift throughout the analytical sequence.

### 2.6 Water quality and phytoplankton analysis

The water temperature (WT) was measured *in situ* with a Yellow Springs instrument (YSI) 6,600 V2 multi-sensor sonde (USA). Total phosphorus (TP-P), soluble reactive phosphorus (SRP-P), and ammonia (NH_4_^+^-N) nitrate (NO_3_^−^-N) concentrations were analyzed according to the technical specifications requirements for monitoring of surface water and waste water in China (HJ/T91–2002).

For phytoplankton analysis, the water samples were fixed immediately with 1% acidic Lugol’s solution after collection to preserve phytoplankton cells. Phytoplankton species were identified and counted according to Hu and Wei (2006). More than 100 individuals for each slide were counted under a light microscope (Eclipse TE200, Nikon, Japan) at 400 × magnification.

Species density (cells/L) was calculated by Equation 1:

$$D=\frac{N\times \mathrm{Vs}}{\mathrm{Vc}\times \mathrm{Vf}}\times 100\%\;\;$$ (1)

Where D is density, N is the counted cell number, *Vs* is the settled volume (50 mL), Vc is the counting chamber volume (0.1 mL), and Vf is the filtered water volume (1 L). Dominant species were defined as those with relative abundance ≥ 50%.

### 2.7 Statistical analysis

The differences in microcystin concentrations determined by three methods in each lake were evaluated using one-way ANOVA in IBM SPSS Statistics 26 software. Significant differences among the three methods were evaluated using the LSD t-test (*p* < 0.05). Spearman’s correlation analysis was conducted to assess the relationship between the proportion of MC congeners and the corresponding MC-LR equivalents measurement error, based on data from the seven lakes. Additionally, Spearman’s correlation analysis was conducted between the density of cyanobacteria species and the physico-chemical indicators of Bear and Muskegon Lake, using IBM SPSS Statistics 26.

The contribution of MC congeners (MC-YR, MC-RR, and other MC congeners except MC-LR, MC-YR, and MC-RR) to MC-LR equivalents measurement error by PPIA or ELISA was analyzed by Generalized additive models (GAMs), with the “mgcv” package in R version 4.2.2. The proportion of each MC congener to total MCs was calculated according to Equation 2, as follows:

$$\text{Proportion of}\;\mathrm{MC}\;\text{congener}=\frac{\begin{array}{c} \mathrm{MC}\;\text{congener}\\ \;\text{concentration} \end{array}}{\begin{array}{c} \text{Total}\;\mathrm{MCs}\;\\ \text{concentration}\; \end{array}}\times 100\%\;$$ (2)

Where Total MCs concentration represented the sum of these three microcystin congeners (MC-LR, MC-YR, and MC-RR). Given that the primary microcystin congeners in lake samples are MC-LR, MC-YR, and MC-RR. The measurement error is defined as the percentage of MC-LR measurement difference between ELISA/PPIA and HPLC/MS.

MC-LR equivalents measurement error was calculated by the Equation 3:

$$\text{Measurement error}=\frac{C_{1}-C_{2}}{C_{2}}\times 100\%$$ (3)

Where *C_1_* represented the MC concentration measured by PPIA or ELISA (ng/L), and *C_2_* represented the MC-LR concentration measured by HPLC/MS (ng/L). The GAM analysis then used the proportion of MC congeners and corresponding MC-LR equivalents measurement error by ELISA and PPIA. Deviance explained, *p*-values and adjusted R^2^ values were used to identify MC congeners that significantly influence MC-LR quantification.

## 3 Results

### 3.1 Microcystin concentrations in seven lakes

Microcystin (MC) concentrations varied substantially among the seven drowned river mouth lakes, reflecting strong spatial heterogeneity linked to trophic status (Table 1). The highest toxin levels occurred in the hypereutrophic systems—particularly Bear Lake, Lake Macatawa, and Muskegon Lake—where dense cyanobacterial blooms were observed. In contrast, mesotrophic to eutrophic lakes such as Duck Lake and White Lake exhibited consistently low MC levels.

**Table 1:** The dominant species and the mean microcystin concentrations of the seven lakes.

Lakes	PPIA (ng/L)	ELISA (ng/L)	HPLC/MS (ng/L)
Bear	4,592 ± 8,558	2064 ± 1,556	MC-LR: 996 ± 1,528
			MC-RR: 984 ± 883
			MC-YR: 156 ± 194
			MC-LA: 10 ± 1
Muskegon	677 ± 579	989 ± 2,600	MC-LR: 896 ± 2,252
			MC-RR: 122 ± 189
			MC-YR: 69 ± 87
			MC-LA: 53 ± 42
Macatawa	973 ± 517	540 ± 246	MC-LR: 191 ± 108
			MC-RR: 2 ± 1
			MC-YR: 6 ± 3
			MC-LA:17 ± 6
White	170 ± 60	110 ± 90	MC-LR: 61 ± 65
			MC-RR: 17 ± 16
			MC-YR: 23 ± 24
			MC-LA: 25 ± 15
Spring	111 ± 25	68 ± 27	MC-LR: 35 ± 14
			MC-RR: 15 ± 6
			MC-YR: 5 ± 4
			MC-LA: 16 ± 12
Mona	157 ± 69	83 ± 47	MC-LR: 54 ± 27
			MC-RR: 5 ± 1
			MC-YR: 3 ± 2
			MC-LA: <1
Duck	75 ± 13	<10	MC-LR: 2.0 ± 0.9
			MC-RR: 0
			MC-YR: 0
			MC-LA: 0

ELISA and PPIA assays report MC-LR equivalents, reflecting the sample’s overall immunoreactive or inhibitory microcystin signal rather than the concentration of a single congener. In contrast, HPLC/MS quantifies individual congeners (MC-LR, MC-YR, MC-RR, and MC-LA), with the total HPLC/MS value representing the sum of these four compounds. Together, these methods provide complementary—but not identical—indicators of total microcystins: ELISA and PPIA offer assay-level estimates, while HPLC/MS delivers precise quantitative measurements of specific congeners.

In the hypereutrophic lakes, PPIA consistently yielded the highest values. In Bear Lake, the mean MC-LR–equivalent concentration by PPIA was 4,592 ± 8,558 ng/L (range: 1360–48,131 ng/L), with a shoreline scum maximum of 48,131 ng/L. ELISA reported a mean of 2064 ± 1,556 ng/L (range: 780–9,080 ng/L), while HPLC/MS detected a total of 2,136 ± 2,583 ng/L (sum of MC-LR, MC-YR, MC-RR, and MC-LA). Similar patterns were observed in Muskegon Lake, where average concentrations were 677 ± 579 ng/L (range: <10–1972 ng/L; PPIA), 989 ± 2,600 ng/L (range: 16–14,826 ng/L; ELISA), and 1,088 ± 2,527 ng/L (range: 7–14,175 ng/L; HPLC/MS).

In the low-toxin lakes, overall MC concentrations were below 100 ng/L. In Duck Lake, mean concentrations were 75 ± 13 ng/L (range: <10–107 ng/L; PPIA), <10 ng/L (ELISA), and <5 ng/L (HPLC/MS). Mona Lake showed similarly low levels: 157 ± 69 ng/L (range: <10–406 ng/L; PPIA), 83 ± 47 ng/L (range: 20–200 ng/L; ELISA), and 57 ± 29 ng/L (HPLC/MS).

Within 196 samples, 46 samples were not detected by PPIA methods. No cylindrospermopsin or anatoxin-a was detected in any samples (Measurement method was provided in section S2 in Supplementary material).

### 3.2 Comparison of microcystin concentrations measured by PPIA, ELISA, and HPLC/MS

**Figure 2:**
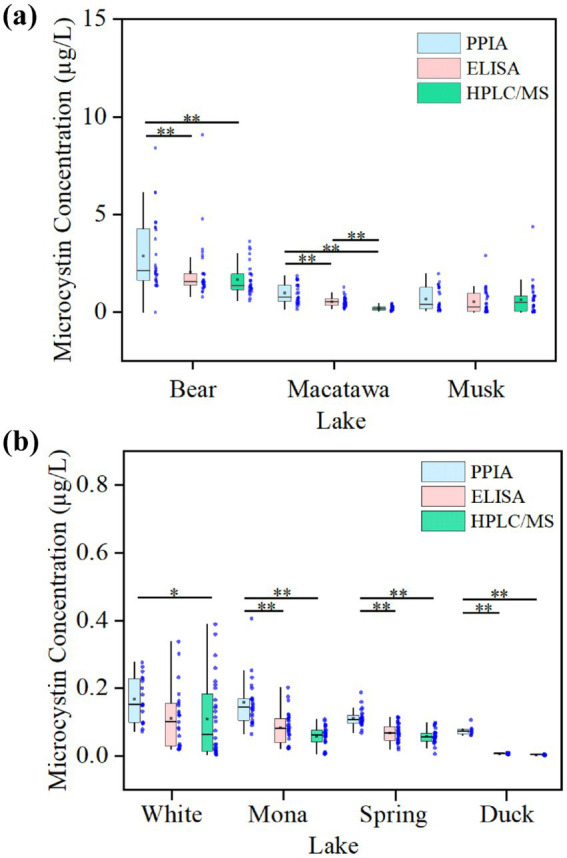
plots of PPIA, ELISA, and HPLC/MS data for seven lakes. * and ** represent significant differences at *p* < 0.05 and *p* < 0.01 levels, respectively. Blue points shows the distribution of data points for microcystin concentration by three methods for seven lakes. For HPLC/MS, measured concentrations of the four congeners are summed to represent a “total” amount of MC.

Microcystin concentrations measured by the three analytical methods (PPIA, ELISA, and HPLC/MS) differed significantly across the seven lakes (Figure 2). Overall, PPIA consistently reported higher concentrations than both ELISA and HPLC/MS. Significant differences between PPIA and the other two methods were observed in Bear Lake, Macatawa Lake, Mona Lake, Spring Lake, and Duck Lake (*p* < 0.01). In White Lake, PPIA results also differed significantly from those of HPLC/MS (*p* < 0.05), while a significant difference between ELISA and HPLC/MS occurred in Macatawa Lake (*p* < 0.01). Except for White Lake, PPIA data exhibited a greater spread than ELISA and HPLC/MS, indicating larger intra-lake variability and reduced measurement consistency.

**Figure 3:**
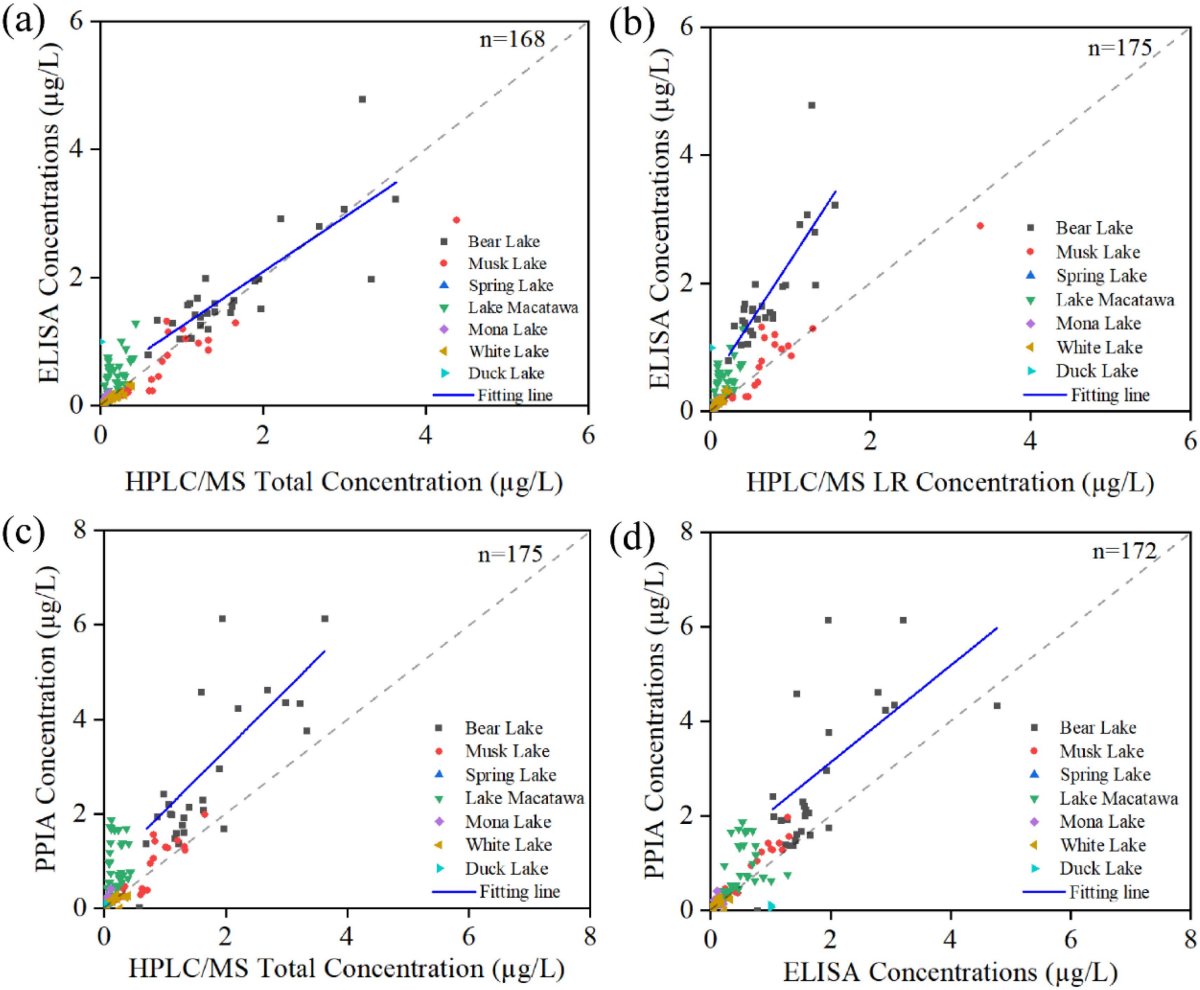
Comparison of MC measurement methods for west Michigan drowned river mouth lakes. **(a)** ELISA versus total microcystins by HPLC/MS, **(b)** ELISA versus MC-LR by HPLC/MS, **(c)** PPIA versus total microcystins by HPLC/MS, **(d)** PPIA versus ELISA. For HPLC/MS, measured concentrations of the four congeners are summed to represent a “total” amount of MC. Dashed line represents a 1:1 relationship.

When comparing ELISA with HPLC/MS-derived total MCs (i.e., the summed concentrations of the four quantified congeners), the regression line closely followed the 1:1 ratio (slope = 0.85, intercept = 0.39, R^2^ = 0.66), except for a few positively deviating points in Macatawa Lake (Figure 3a), suggesting reasonable agreement between the two methods. This deviation likely reflects the broader cross-reactivity of ELISA, which can respond to multiple congeners beyond the four quantified by HPLC/MS. For MC-LR determination, ELISA results generally showed a positive bias relative to HPLC/MS (slope = 1.91, intercept = 0.44, R^2^ = 0.64), with Muskegon Lake as an exception (Figure 3b).

The discrepancy between PPIA and HPLC/MS was even larger (Figure 3c): for total MCs, the regression slope was 1.28 with a low R^2^ = 0.29, indicating consistent higher PPIA response relative to HPLC/MS. Comparison between PPIA and ELISA (slope = 1.02, intercept = 1.08, R^2^ = 0.19) also revealed positive deviation and poor linearity (Figure 3d). These results collectively emphasize that PPIA tends to overestimate toxin concentrations and show greater internal variability, whereas ELISA and HPLC/MS demonstrate better alignment, particularly at moderate to high toxin levels.

### 3.3 Influence of microcystin concentration and congener composition on MC-LR equivalents measurement error in PPIA and ELISA

**Figure 4:**
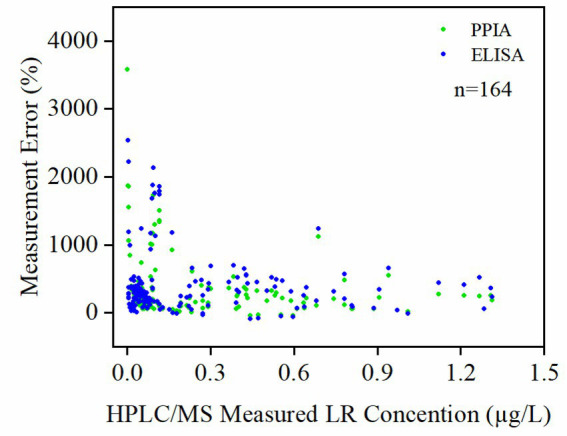
Change of measurement error with MC-LR concentration determined by HPLC/MS. The result used is the measured concentration of the sample.

Comparison of MC-LR equivalent measurement errors between PPIA and ELISA revealed that both assays exhibited decreasing error with increasing toxin concentration, indicating improved accuracy at higher microcystin levels (Figure 4). When HPLC/MS-quantified MC-LR concentration was less than 0.15 μg/L, the average measurement error for PPIA MC-LR equivalents reached 430 ± 456%, significantly exceeding that of ELISA (141 ± 151%, *p* < 0.01). When HPLC/MS-quantified MC-LR ≥ 0.15 μg/L, the mean errors of MC-LR equivalents measurement decreased to 209 ± 211% for PPIA and 103 ± 96% for ELISA, yet PPIA remained significantly higher (*p* < 0.01). Even at moderate to high concentration ranges, occasional spikes in PPIA error were still observed, whereas ELISA maintained more stable performance across the entire range.

To isolate the effect of microcystin congener composition from that of total toxin concentration, correlation and generalized additive model (GAM) analyses were performed using 166 samples above the detection limit (HPLC/MS-quantified MC-LR ≥ 0.15 μg/L) (Table 2). Correlation analysis showed that the proportion of MC-LR (from HPLC/MS quantification) was negatively correlated with measurement errors for both assays, whereas MC-YR (from HPLC/MS quantification) showed a positive correlation with PPIA errors, and MC-RR (from HPLC/MS quantification) was positively correlated with errors in both assays (Table S1).

**Table 2:** Deviance explained, adjusted R^2^ values and *p*-values of generalized additive model incorporating all variables.

Measurement method	ELISA			PPIA		
MC congener proportion	Deviance explained	adjusted R^2^ values	*p*-values	Deviance explained	adjusted R^2^ values	*p*-values
MC-YR	30.70%	0.215	*p*<0.05	69.30%	0.659	*p*<0.01
MC-RR	60.10%	0.577	*p*<0.001	39.30%	0.317	*p*<0.001
other MC congeners	21.20%	0.154	*p*>0.05	42.40%	0.342	*p*<0.01

GAM results further indicated that, for the ELISA method, the proportions of MC-RR and MC-YR significantly influenced measurement error, with adjusted R^2^ values of 0.577 (*p* < 0.001) and 0.215 (*p* < 0.05), explaining 60.1 and 30.7% of deviance, respectively. For the PPIA method, MC-YR exhibited the strongest effect on error magnitude (deviance explained = 69.3%, adjusted R^2^ = 0.659), followed by MC-RR (39.3% and 0.317, respectively). Overall, microcystin congener composition—particularly the relative abundance of MC-YR and MC-RR—plays an important role in shaping the accuracy of MC-LR equivalent estimates obtained by ELISA and PPIA.

### 3.4 Water quality characteristics and their relationship with microcystin

The physicochemical properties and dominant cyanobacterial taxa of the seven lakes are summarized in Table S2. Water temperature (WT) was relatively uniform among lakes, ranging from 24.9 °C to 26.2 °C. Total phosphorus (TP-P) showed pronounced spatial variation, with the highest mean concentration in Muskegon Lake (0.14 ± 0.07 mg/L) and the lowest in Duck Lake (0.01 ± 0.01 mg/L). Soluble reactive phosphorus (SRP-P) remained low in all lakes. Ammonium (NH₄^+^-N) and nitrate (NO₃^−^-N) concentrations also varied, with the highest NH₄^+^-N observed in Muskegon Lake (0.10 ± 0.16 mg/L) and the highest NO₃^−^-N in Spring Lake (0.15 ± 0.10 mg/L). Overall, Muskegon Lake exhibited the highest nutrient levels, reflecting its strong eutrophic status and elevated risk of cyanobacterial proliferation.

Cyanobacterial assemblages were dominated by genera typically associated with eutrophic conditions, primarily *Microcystis* and *Anabaena*. *Microcystis* and *Anabaena* were identified in Bear Lake, Macatawa Lake, White Lake, and Mona Lake, while *Limnothrix* sp.—a common indicator of eutrophication—was detected in Spring Lake and Mona Lake. Duck Lake was dominated by *Aphanocapsa conferta*, *Anabaena flos-aquae*, and *Microcystis wesenbergii*. The predominance of cyanobacteria across these lakes is consistent with their nutrient-enriched conditions.

Because some cyanobacteria are toxin producers, further analysis was conducted for Muskegon Lake and Bear Lake—the two most severely cyanotoxin-affected systems among the seven lakes—to examine relationships among microcystin (MC) congeners, cyanobacterial taxa, and environmental variables (Table 3). In Muskegon Lake, significant negative correlations were observed between nitrate (NO₃^−^-N) and the concentrations of several MC congeners (MC-RR, MC-YR, and MC-LR quantified by HPLC/MS), with MC-YR showing the strongest correlation (r = −0.75). In addition, *Microcystis aeruginosa* (*M. aeruginosa*) was positively correlated with MC-LR concentration (r = 0.44), indicating that this species is a major contributor to MC-LR production in Muskegon Lake.

**Table 3:** Correlation of environmental parameters with different congeners of MC and algae species (*n* = 24 for each parameter).

Lake	Parameters	WT	NO_3_^−^-N	NH_4_^+^-N	SRP-P	TP-P	MC-RR	MC-YR	MC-LR
Muskegon	MC-RR	0.21	−0.55**	0.04	−0.10	0.18	1	0.71**	0.92**
	MC-YR	−0.08	−0.75**	−0.46*	−0.24	−0.14	0.71**	1	0.84**
	MC-LR	0.19	−0.71**	−0.08	−0.06	0.08	0.91**	0.84**	1
	*M. aeruginosa*	0.36	−0.20	0.45*	0.57**	0.30	0.37	0.15	0.44*
	*M. wesenbergii*	0.12	−0.37	−0.25	−0.12	−0.12	0.09	0.32	0.33
Bear	MC-RR	0.04	−0.20	−0.08	−0.08	0.93**	1	0.98**	0.97**
	MC-YR	−0.08	−0.18	−0.01	−0.13	0.96**	0.98**	1	0.99**
	MC-LR	−0.12	−0.18	0.02	−0.10	0.97**	0.97**	0.99**	1
	*M. aeruginosa*	−0.20	−0.23	−0.03	−0.06	0.77**	0.76**	0.75**	0.77**
	*M. wesenbergii*	−0.13	−0.07	−0.15	−0.07	−0.08	−0.01	−0.03	−0.04
	*A. gracile*	0.03	−0.13	−0.08	−0.07	−0.29	−0.27	−0.24	−0.22
	*M. botrys*	−0.35	0.34	0.29	−0.13	−0.04	−0.19	−0.13	−0.12

The * and ** represent significant correlations at 0.05 and 0.01 levels.

In Bear Lake, *M. aeruginosa* was also significantly correlated with all three MC congeners. Total phosphorus (TP) exhibited strong positive correlations with MC-RR, MC-YR, and MC-LR (r = 0.93, 0.96, and 0.97, respectively), suggesting that phosphorus availability is a key driver of toxin production in this system.

## 4 Discussion

### 4.1 Method comparison and analytical interpretation of microcystin quantification

The three analytical methods compared in this study—ELISA, PPIA, and HPLC/MS—interrogate different aspects of the cyanotoxin pool: HPLC/MS yields chemically resolved congener quantification, whereas ELISA and PPIA provide functional estimates of toxin equivalents via immunochemical recognition and phosphatase inhibition, respectively (Tillmanns et al., 2007; Xue et al., 2020). Notably, the MC-LR equivalents reported by ELISA and PPIA represent total immunoreactive or inhibitory microcystin activity and may therefore encompass signals from structurally related congeners or degradation products that share the ADDA moiety or exhibit comparable bioactivity.

Across the seven lakes, the concentration of toxin equivalent to microcystin-LR determined by PPIA was higher than both MC-LR and total MC concentration detected by HPLC/MS, except in the case of Muskegon Lake. The higher PPIA response likely reflects the integration of multiple protein phosphatase inhibitors present in natural water, including other MC congeners and unrelated compounds such as okadaic acid, tautomycin, and calyculin A (Kumar et al., 2020; Mountfort et al., 2005). The weaker MC quantification stability of PPIA compared to ELISA can be explained by significant limitations due to variations in enzyme purity and instability of enzyme dimers in samples (Jaramillo and O’Shea, 2018). This is further supported by the greater dispersion of data points observed for PPIA compared to ELISA and HPLC/MS.

In addition, ELISA consistently yielded higher MC-LR concentrations than HPLC/MS across the seven lakes, a well-documented phenomenon in previous studies (Conti et al., 2006; Tillmanns et al., 2007). This is generally attributed to ELISA’s broader cross-reactivity with microcystin congeners and ADDA-containing degradation products, compared with the congener-specific detection of HPLC/MS (Baliu-Rodriguez et al., 2022; Guo et al., 2017; Kumar et al., 2020; Preece et al., 2021; U.S. EPA, 2016a,b). Accordingly, ELISA showed smaller deviations when compared to the summed concentration of the four quantified congeners rather than MC-LR alone.

In addition to microcystin congeners, structurally related toxins such as nodularins—which also contain the ADDA moiety and exhibit strong phosphatase inhibition—may further contribute to the higher apparent concentrations observed in ELISA and PPIA relative to HPLC/MS (Carmichael and An, 1999; Melaram et al., 2024). Because HPLC/MS targets only the quantified microcystin congeners, these additional ADDA-containing compounds would elevate immunochemical and bioassay responses without being reflected in chemical measurements. This mechanism is consistent with the strong correlation observed between ELISA and PPIA deviations (Figure 4) and with the GAM results, which identified congener composition as a significant driver of measurement bias.

By contrast, Muskegon Lake showed strong agreement among the three methods, indicating that MC-LR dominated the toxin pool and accounted for the majority of total microcystins, consistent with prior findings reporting MC-LR comprising 54–87% of total MCs in this lake (Lawrence et al., 2001; Xie et al., 2011). Despite these discrepancies, ELISA results were generally closer to HPLC/MS in lakes dominated by the major congeners (MC-LR, MC-RR, MC-YR, with MC-LA occasionally present), suggesting that antibody cross-reactivity can yield results that more closely align with HPLC/MS when the toxin profile is dominated by a few major congeners. However, at lower toxin concentrations, both immunoassay and bioassay methods tended to yield higher values—likely due to matrix effects and amplified background near detection limits. Collectively, these results indicate that analytical consistency improves in eutrophic systems dominated by common congeners, whereas greater discrepancies emerge in low-toxin or less eutrophic environments, reflecting the broader ecological coupling between nutrient status, bloom intensity, and toxin accumulation across these interconnected coastal lakes.

### 4.2 Factors affecting quantification accuracy

The accurate quantification of MC-LR is essential for risk assessment due to its high biotoxicity. Building upon the methodological differences discussed above, the accuracy is influenced by both analytical limitations and environmental conditions. Among the three techniques, HPLC/MS is generally the most specific and reliable because it can distinguish individual congeners with chemical precision. However, its high cost, technical complexity, and longer analysis time restrict its use for routine monitoring. In contrast, ELISA and PPIA—though unable to differentiate between MC congeners—are widely adopted for their speed, simplicity, and cost-effectiveness, particularly in field-based screening applications. Accordingly, we next evaluated how toxin level and congener composition modulate assay performance.

Our results demonstrate that the measurement accuracy of ELISA and PPIA strongly depends on toxin concentration. When MC-LR equivalents exceeded 0.15 μg/L, the measurement errors of both methods decreased markedly, suggesting improved precision under bloom conditions with elevated toxin levels. This implies that ELISA and PPIA are more appropriate for quantifying microcystins in lakes experiencing active cyanobacterial proliferation, rather than for early warning in low-toxin environments. Across all samples, PPIA exhibited greater deviation than ELISA, suggesting better reproducibility for ELISA under our study conditions. However, PPIA provides a measure of total phosphatase inhibition, representing cumulative bioactivity and toxicity rather than chemical concentration (Carmichael and An, 1999; Zong et al., 2017). Thus, its interpretive value depends on whether the analytical goal is to assess total biological potency or specific toxin mass.

Beyond concentration effects, interference from co-occurring congeners was another major source of quantification error. A higher proportion of MC-LR within the total MC pool reduced measurement uncertainty, whereas elevated proportions of other congeners increased bias, likely due to cross-reactivity and competitive binding in both assays. For ELISA, MC-RR contributed more to the observed error than MC-YR, consistent with the manufacturer’s cross-reactivity coefficients (87% for MC-RR vs. 48% for MC-YR). In contrast, PPIA errors were more strongly influenced by MC-YR, which exhibits a higher inhibitory potency (IC₅₀ = 9 nM) compared to MC-RR (175 nM) and is similar to MC-LR (2.2 nM) (Mountfort et al., 2005). These findings indicate that toxin mixtures with diverse congener compositions can substantially alter quantification outcomes, emphasizing the need for congener-specific interpretation when comparing results across analytical platforms.

### 4.3 Environmental implications and management relevance

The methodological discrepancies identified in this study directly affect environmental risk assessment and management decisions. Higher response of MC-LR equivalents by immunoassays (ELISA and PPIA) may result in conservative management responses, whereas congener-specific quantification by HPLC/MS could underestimate overall toxicity in ecosystems where multiple bioactive congeners co-occur (McElhiney and Lawton, 2005; Hu, 2022). According to U.S. EPA guidelines, the maximum allowable microcystin concentrations are 8 μg/L for recreational waters and 0.3 μg/L for drinking water (U.S. EPA, 2015a,b; U.S. EPA, 2019). Translating our findings into these thresholds suggests that ELISA and PPIA results may occasionally exceed safety limits even when HPLC/MS values remain below them, thereby underscoring the necessity of interpreting data in the context of method-specific biases. Furthermore, 0.15 μg/L is defined as the cutoff for low/high MC load, a value that is lower but close to the 0.3 μg/L threshold set by the EPA. This indicates the limitations of PPIA and ELISA methods for the quantitative analysis of microcystins in relatively clean drinking water sources.

Notably, differences among analytical methods can translate into distinct risk classification outcomes, which in turn influence management actions such as issuing recreational advisories, closing beaches, or initiating water treatment responses. For example, if immunoassay-derived MC-LR equivalents exceed the recreational threshold while HPLC/MS results remain below it, management authorities must balance precautionary closures against maintaining public access. Integrating the results from multiple methods—particularly when interpreting concentrations near regulatory thresholds—can thus prevent both unnecessary restrictions and overlooked risks, improving the reliability and transparency of risk communication.

Beyond analytical precision, these methodological differences have practical management implications. For practical environmental monitoring, a tiered analytical framework is recommended. ELISA serves as a high-throughput screening tool for rapid toxin detection. PPIA is particularly valuable for assessing biological potency and ecological bioactivity, reflecting the overall inhibitory potential of samples. HPLC/MS should be employed for confirmatory quantification and detailed congener profiling. Integrating these complementary approaches enables the development of a scientifically robust yet cost-effective monitoring system that balances sensitivity, specificity, and ecological relevance. This framework enables calibration of rapid field assays against chemical reference methods, enhancing comparability and reliability.

In addition, the tiered monitoring framework can be adapted for regions with limited analytical resources, where simultaneous access to ELISA, PPIA, and HPLC/MS is rarely feasible. In such contexts, a simplified two-tier approach may be more realistic: ELISA can serve as the primary screening tool because of its low cost and operational simplicity, whereas periodic confirmatory analysis using regionally centralized HPLC/MS facilities can provide congener-level validation when resources permit. This resource-sensitive adaptation maintains the benefits of tiered monitoring while acknowledging the practical constraints faced by many countries and local water authorities.

Furthermore, our results underscore a strong ecological linkage between eutrophication and cyanotoxin production. The significant positive correlations observed between total phosphorus and microcystin concentrations highlight nutrient enrichment as a key driver of toxin synthesis. The dominance of *Microcystis aeruginosa* in high-toxin lakes such as Bear and Muskegon further supports this linkage, indicating that nutrient load reduction and continuous bloom surveillance remain central strategies for mitigating cyanotoxin risk in freshwater ecosystems.

Importantly, the value of this study extends beyond a methodological comparison. By systematically reconstructing and reanalyzing a regionally representative multi-lake dataset from the drowned river mouth lakes of western Michigan, this work provides rare, spatially resolved evidence of early cyanotoxin dynamics in the Great Lakes region, with substantial archival and reference significance. Through the reanalysis of historical samples, this study contributes valuable insight into the long-term dynamics of microcystins and the evolution of monitoring practices, providing a useful reference for future environmental risk assessment and water management efforts.

## 5 Limitations and future perspectives

The identified limitations primarily reflect the historical and methodological context of the dataset rather than flaws in the study design. This analysis focused primarily on particulate-phase microcystins retained on filters, as dissolved-phase data were not available in the original records. Because most health-risk assessments, including those by the U.S. EPA, are based on total microcystin concentrations (particulate + dissolved), the fact that this study analysed only the particulate phase may lead to an underestimation of absolute exposure potential (U.S. EPA, 2015a, 2015b; U.S. EPA, 2019). Nevertheless, focusing on the particulate fraction still yields valuable insight into the spatial patterns and ecological distribution of microcystins associated with cyanobacterial biomass, which are key indicators for bloom dynamics and toxin accumulation processes.

HPLC/MS quantification requires certified analytical standards for each congener to achieve accurate measurement. However, many microcystin congeners lacked commercially available standards at the time of sampling in 2006, which limited the number of variants that could be reliably quantified. Consequently, only four congeners (MC-LR, MC-RR, MC-YR, and MC-LA) were analyzed in this study. Although this restricted the overall congener coverage and may have led to an underestimation of total microcystin concentrations, these four congeners are widely reported as dominant variants in North American waters (Xie et al., 2011). Therefore, they still provide a representative basis for inter-method comparison and environmental interpretation. In addition, in complex environmental samples, matrix effects may influence ionization efficiency and thus affect analytical accuracy. The absence of dedicated matrix spike samples in the original dataset limited our ability to fully evaluate matrix-dependent bias. Incorporating matrix spike experiments and/or additional surrogate standards in future studies would strengthen method validation and improve the assessment of matrix effects.

The dataset was derived from 2006 field samples, reflecting the analytical capabilities and methodological practices of that time. Cyanotoxin assay standardization was still evolving, and consistent long-term datasets were limited. Although some procedural details were unavailable, extensive efforts were made to validate and restore the historical dataset, ensuring the reliability and comparability of the analysis. Differences in calibration—MC-LR–based standards for ELISA and PPIA versus multi-congener quantification in HPLC/MS—may have contributed to the observed discrepancies, highlighting the continued need for unified reference materials and cross-method harmonization in future monitoring work.

Despite these constraints, this study provides unique value by revisiting a regionally representative multi-lake dataset and applying modern analytical perspectives to interpret early cyanotoxin data. Such retrospective analyses offer critical baseline insights into the occurrence, variability, and monitoring evolution of microcystins in the Great Lakes region. Future research should integrate high-resolution mass spectrometry with bioassays to connect chemical identity and biological potency, expand congener coverage, and include both particulate and dissolved toxin fractions. Combining toxin profiles with cyanobacterial genomics and nutrient dynamics will enhance ecological risk frameworks and predictive bloom models. Additionally, the development of accurate, cost-effective detection tools remains essential for routine surveillance. Strengthening bloom monitoring and nutrient control remains essential for mitigating microcystin production (Ai et al., 2020). These actions are central to safeguarding freshwater ecosystems and public health.

## Data Availability

The raw data supporting the conclusions of this article will be made available by the authors, without undue reservation.
