# Supplementary material for: Comparative evaluation of ELISA, PPIA, and HPLC/MS for microcystin quantification in seven lakes of western Michigan
Source: Front Microbiol. 2026 May 21;17:1733970. doi: 10.3389/fmicb.2026.1733970 (PMC13233501; doi:10.3389/fmicb.2026.1733970)
Supplement: Supplementary file 1 [file Supplementary_file_1.DOCX]

**Supplementary Information**

**Comparative evaluation of ELISA, PPIA, and HPLC/MS for microcystin quantification in seven lakes of western Michigan**

Chen Cheng^a^, Xiaonan Tang^d^, Yongjiu Cai^a^, Miao Jin^a^, Dailan Deng^a^, Liqiang Xie^b*^, Richard R. Rediske^c*^

^a^State Key Laboratory of Lake and Watershed Science for Water Security, Nanjing Institute of Geography and Limnology, Chinese Academy of Sciences, Nanjing 211135, China

^b^School of Ecology and Environment, Anhui Normal University, Wuhu, Anhui 241002, China

^c^Annis Water Resource Institute, Grand Valley State University, 740 West Shoreline Drive Muskegon, MI 49441, United States

^d^Department of Civil and Environmental Engineering, The George Washington University, Washington, District of Columbia 20052, United States

**Section S1** **Effects of 5% methanol on MC-LR concentration measurement**

We evaluated whether residual methanol in sample affected MC quantification. The stock solution of MC-LR was obtained by adding MC-LR standard (Sigma-Aldrich, München, Germany) to deionized water. The MC-LR stock solution was added in deionized water and in 5% (*v/v*) methanol respevtively to obtain solution with MC-LR concentrations of 2 µg/L, and their concentrations were determined using ELISA kit and PPIA method. Differences within each analytical method were assessed using an LSD t-test (p < 0.05). No significant difference was observed between the control and the 5% methanol treatments (Fig. S1), indicating that methanol at this concentration does not interfere with the assays.


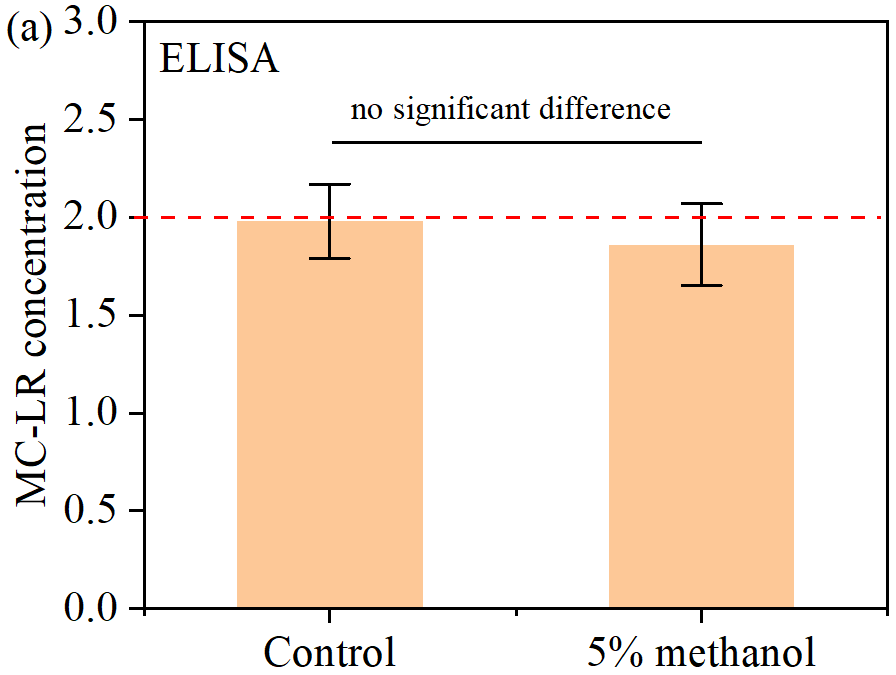

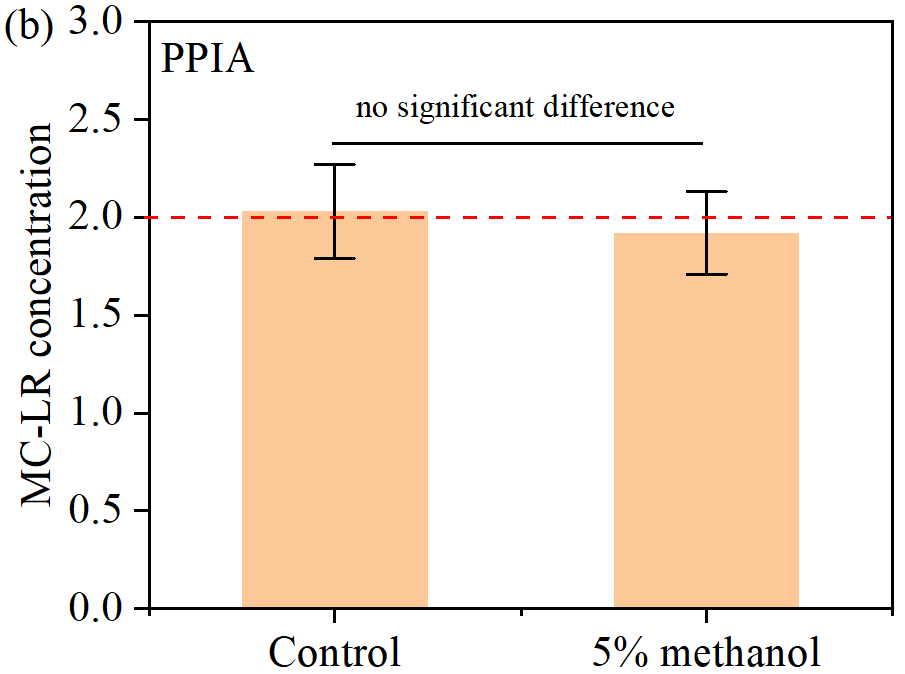


**Fig.S1** Effects of 5% methanol on MC-LR concentration measured by (a) ELISA and (b) PPIA

**Section S2 Determination of anatoxin-a and cylindrospermopsin by LC/MS/MS**

The concentrations of cylindrospermopsin and anatoxin-a were determined by a Waters Quattro Micro LC/MS/MS (Milford, USA). Nodularin was added to the extracts and used as the internal standard. Compounds were separated on a Betabasic C18 column (Thermo, Massachusetts, USA) at 50℃. The mobile phase was a binary gradient of water and methanol, both containing 0.1% formic acid. The mobile phase gradient elution procedure was as follows: 95% water and 5% methanol during 0-3 min; the proportion of methanol increased from 5% methanol to 50% during 3-5 min; then the proportion of methanol increased 50% to 95% during 5-20 min. The instrument detection limits for these toxins were determined to be near 20 picograms on column. For calibration, a series of 6 solutions were prepared with the internal standard at 1000 pg/μL and the analytes in the range of 1 to 500 ng/L in final volumes of 1 ml of 90:10 water: methanol (v/v).

**Tab S1 Correlation results between concentrations of MC congeners and MC-LR measurement error of PPIA and ELISA methods**

| Measurement methods | PPIA | ELISA |
| --- | --- | --- |
| MC-LR proportion | -0.485** | -0.488** |
| MC-YR proportion | 0.286* | 0.191 |
| MC-RR proportion | 0.521** | 0.525** |
| other MC congeners proportion | -0.108 | -0.118 |

* and ** indicates a significant difference between two variables at *p*<0.05 and *p*< 0.01, respectively.

**Table S2 Water quality characteristic and dominant cyanobacterial species**

| Lakes | WT  (°C) | TP-P  (mg/L) | SRP-P  (mg/L) | NH_4_^+^-N  (mg/L) | NO_3_^-^-N  (mg/L) | Dominate cyanobacterial species |
| --- | --- | --- | --- | --- | --- | --- |
| Bear | 26.06±2.46 | 0.06±0.09 | 0.01±0.00 | 0.03±0.02 | 0.03±0.01 | *Aphanizomenon gracile*  *Microsystis aeruginosa*  *Microsystis botrys*  *Microsystis viridis* |
| Muskegon | 25.04±1.40 | 0.02±0.01 | 0.01±0.00 | 0.05±0.08 | 0.10±0.04 | *Microsystis aeruginosa Microsystis wesenbergii* |
| Macatawa | 25.17±1.40 | 0.14±0.07 | 0.01±0.01 | 0.10±0.16 | 0.12±0.12 | *Microsystis aeruginosa Planktothrix argardhii Planktothrix rubsescens Anabeana flos-aquae* |
| White | 24.86±1.56 | 0.03±0.01 | 0.01±0.00 | 0.04±0.02 | 0.05±0.04 | *Microsystis wesenbergii Anabeana flos-aquae Anabeana mendota Microsystis aeruginosa* |
| Spring | 25.72±0.78 | 0.03±0.01 | 0.01±0.01 | 0.06±0.05 | 0.15±0.10 | *Limnothrix sp.* |
| Mona | 25.38±1.16 | 0.06±0.04 | 0.01±0.00 | 0.03±0.02 | 0.03±0.03 | *Anabeana flos-aquae Limnothrix sp. Aphanizomenon gracile Microsystis aeruginosa* |
| Duck | 26.15±1.75 | 0.01±0.01 | 0.01±0.00 | 0.02±0.01 | 0.01±0.00 | *Ahanocapsa conferat*  *Anabeana flos-aquae*  *Microsystis wesenbergii* |

The values in the table are mean ± standard deviation
